# Host-induced climate change: Carbon dioxide tolerance as a *Cryptococcus neoformans* virulence trait

**DOI:** 10.1371/journal.ppat.1013351

**Published:** 2025-08-08

**Authors:** Emma E. Blackburn, Laura C. Ristow, Xiaorong Lin, Damian J. Krysan

**Affiliations:** 1 Department of Microbiology, University of Georgia, Athens, Georgia, United States of America; 2 Department of Pediatrics, Carver College of Medicine, University of Iowa, Iowa City, Iowa, United States of America; 3 Department of Plant Biology, University of Georgia, Athens, Georgia, United States of America; 4 Department of Molecular Physiology and Biophysics, Carver College of Medicine, University of Iowa, Iowa City, Iowa, United States of America; Geisel School of Medicine at Dartmouth, UNITED STATES OF AMERICA

## What features of host physiology must environmental fungi overcome to cause disease in mammals?

Fungi such as *Cryptococcus neoformans*, *Histoplasma capsulatum*, *Blastomyces dermatitidis*, and *Coccidioides* spp. are important human fungal pathogens which naturally exist in the environment [1]. Consequently, these fungi must transition from their natural ecological niches to the host environment in order to cause disease in humans. The elevated body temperature of mammals is a profound physiologic bottleneck that reduces the pathogenic potential of the great majority of the world’s species of fungi [2]. In *C. neoformans*, for example, the ability to tolerate human body temperature is a well-established and intensively studied virulence trait that distinguishes many non-pathogenic environmental strains from those isolated from patients [3]. However, recent studies of large *C. neoformans* clinical isolate collections indicate that differences in temperature tolerance or other virulence traits such as polysaccharide capsule formation and cell wall melanization cannot comprehensively account for the observed variation in mammalian virulence [4]. One aspect of the mammalian host environment that is dramatically different from the external environment is carbon dioxide concentration: ambient air contains approximately 0.04% CO_2_ while mammalian tissues contain 100-fold higher concentrations (~5%). We hypothesized that this dramatic difference may represent a significant stress for *C. neoformans* and, indeed, competitive growth assays demonstrated that clinical isolates are, generally, more tolerant of host CO_2_ concentrations than strains isolated from the environment [5,6]. Furthermore, CO_2_-intolerant environmental strains were less virulent than CO_2_-tolerant environmental strains [5].

## What aspects of *C. neoformans* physiology are affected by host CO_2_ concentrations?

**Fig 1:**
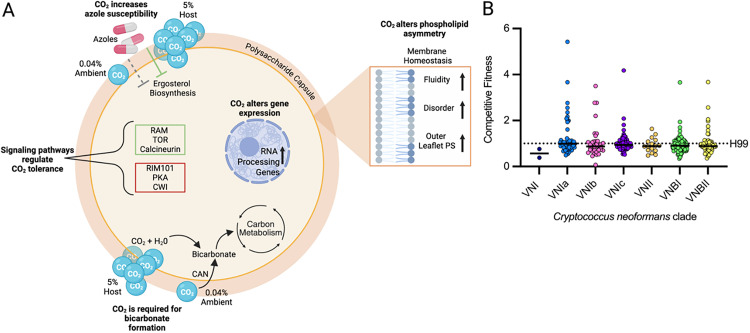
Carbon dioxide tolerance as a virulence trait in *Cryptococcus neoformans.* **A.** Schematic summarizing the effects of CO_2_ on *C. neoformans* cellular physiology and the signaling pathways that positively (green box) and negatively (red box) CO_2_ tolerance. Carbonic anhydrase (CAN). **B.** The competitive fitness of environmental and clinical isolates from the indicated *C. neoformans* clades in 5% CO_2_ in buffered RPMI medium at 37°C relative to the reference strain H99; data are from reference [6].

CO_2_ plays an essential role in cellular physiology through its conversion to bicarbonate which, in turn, is a critical substrate for multiple reactions involved in central carbon metabolism (Fig 1A). In the low CO_2_ concentrations of the external environment, *C. neoformans*, like other fungi, generates bicarbonate from CO_2_ and water through the enzyme carbonic anhydrase [7]. In the host, the high concentrations of CO_2_ drive spontaneous bicarbonate formation. As a result, *C. neoformans* carbonic anhydrase, which is essential in ambient air, is dispensable during mammalian infection [7]. The generation of bicarbonate releases a proton, but our work has shown that the effects of CO_2_ on *C. neoformans* are independent of changes in pH and are not recapitulated by changes in bicarbonate concentration [5].

High CO_2_ concentrations cause cellular stress in fungi and bacteria. Indeed, this stress is so significant that it has been exploited in the food industry, where high CO_2_ atmospheres are used to prevent food spoilage by bacterial and/or fungal growth (modified atmosphere packaging). Studies on the effects of high CO_2_ exposure indicate it alters the ratio of unsaturated/saturated fatty acids of bacterial and fungal membranes and leads to a more fluid phospholipid bilayer and causes membrane disorder [8]. Consistent with these studies, we found that the phospholipid asymmetry of *C. neoformans* plasma membrane is remodeled at host CO_2_ concentrations [9]. Specifically, phosphatidylserine, which is normally localized to the inner leaflet of the plasma membrane, accumulates on the outer leaflet (Fig 1A). *C. neoformans* strains expressing high levels of an ABC transporter (*PDR9*) that interferes with phospholipid remodeling of the plasma membrane are less fit in CO_2_.

In addition, our group has shown that the susceptibility of *C. neoformans* to azole drugs, which target synthesis of the fungal membrane sterol, ergosterol, is increased at host concentrations of CO_2_. Although host CO_2_ appears to have a profound effect on membrane homeostasis, our group has shown that CO_2_ tolerance is a quantitative trait with multiple genetic loci contributing to the genetic basis for the difference between CO_2_-tolerant and sensitive strains [10]. Current studies are under way to understand how other aspects of C. neoformans physiology are affected by host concentrations of CO_2_ stress.

## What stress response pathways affect *C. neoformans* CO_2_ tolerance?

Our large-scale genetic screens of publicly available transcription factor and protein kinase deletion mutant collections [9,11] have identified three pathways that positively regulate *C. neoformans* CO_2_ tolerance: (**1**) Target of Rapamycin (TOR); (**2**) Regulator of Ace2 Morphogenesis (RAM), and (**3**) calcineurin—that positively regulate CO_2_ tolerance in *C. neoformans*. Conversely, the Cell Wall Integrity (CWI), Rim 101, and protein kinase A (PKA) pathways negatively regulate CO_2_ tolerance (Fig 1A). The distinct roles of these important regulators of *C. neoformans* physiology suggest that CO_2_ stress tolerance requires a balanced response from multiple stress-related pathways.

To date, we have explored the specific functions of the TOR and RAM pathways during CO_2_ stress. The TOR pathway, through its target kinase Ypk1, plays a critical role in membrane homeostasis, particularly with respect to membrane rigidity/fluidity and sphingolipid biosynthesis [12]. The latter process is critical to CO_2_ tolerance as demonstrated by the increased susceptibility of *C. neoformans* to the sphingolipid biosynthesis inhibitor myriocin in 5% CO_2_ [5]. Furthermore, the TOR-Ypk1 pathway plays a critical regulatory role in remodeling phospholipid asymmetry in response to CO_2_. Previous work in *C. neoformans* has also shown that the TOR pathway suppresses CWI pathway activation [13]. Consistent with this function, we demonstrated that host CO_2_ blunts temperature-induced CWI activity, providing an explanation for the increased CO_2_ fitness of deletion mutants lacking CWI pathway kinases [9].

The RAM pathway has been extensively characterized in *Saccharomyces cerevisiae* and regulates cell wall integrity, daughter cell-specific gene expression, mating, polarized growth, RNA turnover, and stress signaling [14]. However, the functions of the RAM pathway in ascomycetes (e.g., *S. cerevisiae*) are distinct from basidiomycetes (e.g., *C. neoformans*). For example, RAM pathway mutants in ascomycetes have reduced abilities to undergo polarized growth, whereas *C. neoformans* RAM mutants display a hyperpolarized morphology [15]. To determine which function(s) of the RAM pathway are related to CO_2_ tolerance, we isolated two groups of mutants of strains lacking *CBK1*, the key RAM pathway kinase, that had regained the ability to grow at 5% CO_2_. Both suppressor strains contained mutations in genes involved in RNA homeostasis: *SSD1*, an RNA binding protein known to be a direct substate of Cbk1 in multiple other fungal species [16], and *PSC1*, an uncharacterized gene that contains a Poly(A)-specific ribonuclease (PARN) domain [11]. Transcriptional profiling of *C. neoformans* revealed that RNA processing genes are significantly enriched in the set of genes differentially expressed in 5% CO_2_ [9]. Thus, it appears that at least one RAM pathway function important for CO_2_ tolerance is the regulation of RNA homeostasis.

## What is the relationship between CO_2_ tolerance and *C. neoformans* virulence?

We initially found a strong correlation between CO_2_ tolerance and virulence by determining the CO_2_ fitness of a set of environmental and clinical *C. neoformans* strains whose virulence in a mouse model of cryptococcosis had been characterized by Litvintseva and Mitchell [5,17]. We have also used bulk segregant analysis combined with fine mapping of near-isogenic progeny from crosses of CO_2_-tolerant and -intolerant strains to show that virulence strongly correlated with CO_2_ fitness [10]. Infection of mice with a CO_2_-intolerant *C. neoformans* strain led to mortality in a minority of mice. Importantly, strains isolated from these mice were now CO_2_-tolerant, suggesting that the strains were able to adapt to host CO_2_ during infection [10]. Finally, in vitro microevolution of CO_2_-sensitive strains in a high CO_2_ environment generated mutants with increased CO_2_ fitness [6]. Two of these evolved, CO_2_-tolerant strains contained truncation mutations in *AVC1*, an ARID domain-containing protein thought to be involved in transcriptional regulation [16]. Deletion of *AVC1* in multiple CO_2_-sensitive strains led to increased CO_2_ fitness and virulence in mice. Intriguingly, similar loss-of-function mutations in *AVC1* have been found in *C. neoformans* strains isolated from patients with relapsed cryptococcosis [18,19]. Together, these data strongly support the idea that CO_2_ tolerance is virulence trait and also suggest that CO_2_ stress exerts a selective pressure during infection of both mice and humans.

## How is CO_2_ tolerance distributed among the clades of *C. neoformans*?

As mentioned above, we have recently characterized the CO_2_-tolerance of a large set of clinical and environmental isolates [9]. This set of isolates contained strains from VNI, VNIa, VNIb, VNIc, VNBI, VNBII, and VNII (Fig 1B). Both CO_2_-tolerant and -sensitive strains were found in all clades with no one clade showing a significantly increased or decreased number of CO_2_-tolerant or -intolerant strains. Within a given clade, we also found that many highly related strains showed different CO_2_ fitness. This distribution pattern suggests that CO_2_ tolerance may have emerged many times during the evolution of *C. neoformans* rather than having originated in a specific lineage. Our quantitative trait loci analyses indicate that many genetic loci contribute to CO_2_ tolerance. Thus, it seems likely that multiple potential genetic and molecular mechanisms could drive the emergence of CO_2_ tolerance in any given strain; the exact nature of these mechanisms is an open question that we are currently investigating. It is also interesting to consider what niche or environmental conditions leads to the selection of CO_2_ tolerance? What niche selects against CO_2_ tolerance in environmental strains? And finally, do latent infections provide a niche under which *C. neoformans* adapts to the host environment and, thereby, increases its fitness as a pathogen?
